# Microaneurysm detection in fundus images using a two-step convolutional neural network

**DOI:** 10.1186/s12938-019-0675-9

**Published:** 2019-05-29

**Authors:** Noushin Eftekhari, Hamid-Reza Pourreza, Mojtaba Masoudi, Kamaledin Ghiasi-Shirazi, Ehsan Saeedi

**Affiliations:** Machine Vision Lab., Computer Engineering Department, Faculty of Engineering, Ferdowsi University of Mashhad (FUM), Azadi Sqr., Mashhad, Iran

**Keywords:** Diabetic retinopathy (DR), Microaneurysm (MA), Deep learning, Convolutional neural network (CNN)

## Abstract

**Background and objectives:**

Diabetic retinopathy (DR) is the leading cause of blindness worldwide, and therefore its early detection is important in order to reduce disease-related eye injuries. DR is diagnosed by inspecting fundus images. Since microaneurysms (MA) are one of the main symptoms of the disease, distinguishing this complication within the fundus images facilitates early DR detection. In this paper, an automatic analysis of retinal images using convolutional neural network (CNN) is presented.

**Methods:**

Our method incorporates a novel technique utilizing a two-stage process with two online datasets which results in accurate detection while solving the imbalance data problem and decreasing training time in comparison with previous studies. We have implemented our proposed CNNs using the Keras library.

**Results:**

In order to evaluate our proposed method, an experiment was conducted on two standard publicly available datasets, i.e., Retinopathy Online Challenge dataset and E-Ophtha-MA dataset. Our results demonstrated a promising sensitivity value of about 0.8 for an average of >6 false positives per image, which is competitive with state of the art approaches.

**Conclusion:**

Our method indicates significant improvement in MA-detection using retinal fundus images for monitoring diabetic retinopathy.

## Introduction

Diabetes mellitus (DM), commonly referred to as diabetes, is a growing disease in the world. According to the World Health Organization (WHO) statistics, it is predicted that the number of people having DM will reach 439 million by 2030. One of the main complications of DM is diabetic retinopathy (DR) which is one of the most serious diseases of the eye and one of the main causes of blindness in the world. Hence, accurate and early diagnosis of this disease can prevent the development of blindness. Detection of DR is done by examination of fundus and optical coherence tomography (OCT) images [1, 2].

Microaneurysms (MA) is usually the first symptom of DR that causes blood leakage to the retina. This lesion usually appears as small red circular spots with a diameter of fewer than 125 micrometers [3]. Therefore, periodic screening and detection of MA will result in early detection of DR and reduction of eye injuries. However, screening and timely re-screening of DR is time-consuming and very costly [4]. As a result, many research has been conducted on analytical techniques and the automatic identification of MA. Although, OCT has become a powerful imaging modality for diagnosis of various DR abnormalities, most of the CAD systems for early MAs detection use fundus images of the patient. In this study, we also use fundus images due to lack of available OCT dataset for detecting MAs.

Artificial neural networks and deep learning, conceptually and structurally inspired by neural systems, rapidly become an interesting and promising methodology for researchers in various fields including medical imaging analysis. Deep learning means learning of the representations of data with multiple levels of abstraction used for computational models that are composed of multiple processing layers. These methods rapidly become an interesting and promising methodology for researcher and are gaining acceptance for numerous practical applications in engineering [5]. Deep learning has performed especially well as classifiers for image-processing applications and as function estimators for both linear and non-linear applications. Deep learning recognizes complicated structure in big datasets by utilizing the back propagation algorithm to indicate how the internal parameters of a NN should be changed to compute the representation in each layer from the representation in the previous layer [6].

In particular, convolutional neural networks (CNNs) automatically learn mid-level and high-level abstractions obtained from raw data (e.g., images), and so have been considered as powerful tools for a broad range of computer vision tasks [6]. Recent results indicate that the generic descriptors extracted from CNNs are extremely effective in object recognition and localization in natural images [6]. Also, Medical image analysis is quickly entering the field and applying CNNs and other deep-learning methodologies to a wide variety of applications [5, 6].

Problems such as poor image quality, differences in the size of MAs, the closeness of some MAs to the vessels, and the low number of pixels belonging to MAs, which themselves generate an imbalanced data in the learning process, have caused many MA-detection algorithms to provide low accuracy results. Consequently, MA-detection is still among the open issues. In this study, we propose to take advantage of deep learning especially convolutional neural networks to tackle with the above challenges by increasing the accuracy of MA-detection and addressing imbalanced data in fundus images.

### Related work

There are multiple approaches developed by the research community in the area of automated MA-detection CAD system. In these techniques, firstly, the quality of the image is improved by pre-processing the input image. This pre-processing step includes contrast enhancement [7, 8], shade correction [9], noise elimination [7], and in some cases, removal of anatomical components such as the bright lesion and vessels [10]. Then the identification of MAs is done on the resulting image. Various methods are used for this purpose including mathematical morphology techniques, template matching techniques, pixel classification methods, and hybrid methods. Early techniques for MA identification are generally based on the use of mathematical morphology on fluorescein angiography images [3, 11–13]. In these papers, vessel removal is done by employing directional structural elements in various directions and then using the top-hat transform. The hit-or-miss transform is also another approach in this category which is used in [14]. The second category of techniques for finding MA candidates is template matching using different filters such as Gaussian filter [10, 15–17] and a double-ring filter [18]. In these methods, Gaussian kernel size is chosen empirically and hence, changing the size of MAs can reduce the performance of these algorithms. Thresholding [19–21], the feature extraction based on Hessian matrix property [22], the extended minima transform [23, 24], and the wavelet transforms [25] are methods that are in the third category of MA identification techniques, pixel classification based methods. In these methods, linear discriminant analysis(LDA), k-nearest neighbors algorithm(KNN) [8, 15, 17], artificial neural network [14, 21], Navie Bayse [23] are different classifiers which are employed. Also, in some articles, unsupervised learning methods such as mixture model (MM) clustering are used. Despite the fact that there is no need for training data, these methods cannot compete with the supervisor’s learning methods [7, 9, 16, 19, 20, 26]. Furthermore, examples of hybrid techniques, as the fourth category of MA identification methods, have been reported in [12, 15, 16].

A various method has been proposed by using deep neural networks. A stacked sparse auto-encoder (SSAE) an instance of a deep-learning method is proposed by Shan et al. [27]. This method can be built by incorporating multiple layers of sparse auto-encoder. The SSAE learns high-level features of MA. The high-level features learned by SSAE are fed into a softmax classifier to distinguish between MA or non-MA image patches. Budak et al. [28] presented a three stages includes pre-processing, five-stepped procedure to detect potential MA locations and deep convolutional neural network (DCNN) with reinforcement sample learning strategy to classify MA and non-MA. Later, Chudzik et al. [29] used a patch-based fully CNN which provided a novel network fine-tuning scheme called Interleaved Freezing. They claimed that the re-train time is reduced. The method by Cirecsan et al. [30] for mitosis detection on histopathology images is also similar to ours. It uses candidate detection as well, using a simplified version of the boosting strategy is a two-step approach in which misclassified samples of an initial model are used as the training set of a second independent learner.

Recently, researchers are studying to define more robust reference standards that can be used to quantify performance. They use a 3D imaging technology, optical coherence tomography (OCT), to examine various layers of a retina in detail. ElTanboly et al. [31] proposed a CAD system for detecting DR in OCT images. In the first stage they localize and segment the retinal layers by Markov-Gibbs random field (MGRF) model and then extract features from segmented layers. Finally they used deep fusion classification network (DFCN) to classify normal or diabetic regions. Sandhu et al. [2] presented a novel CAD system that segments the retina into 12 layers and then some global features such as curvature, reflectivity, and thickness measured. Finally, a two-stage, deep network is used to classify normal and abnormal areas. Although, OCT has become a powerful imaging modality for diagnosis of various DR abnormalities. However, most of the CAD systems for early microaneurysms detection use fundus images of the patient. In the future, using these two complementary methods can be used together also to detect MAs with more precision.

### Contribution

In this paper a new method for MA-detection in fundus images based on deep-learning neural networks is developed to overcome the problems of the current automatic detection algorithms. Also, only few papers directly address issues specific to object detection like class imbalance/hard-negative mining or efficient pixel/voxel-wise processing of images. We expect that more emphasis will be given to those areas in the near future, for example in the application of multi-stream networks in a fully convolutional fashion [32, 33].

Deep-learning algorithms and in particular, convolutional networks, have rapidly become a methodology of choice for analyzing medical images [13]. Deep learning is an improvement of artificial neural networks with more layers which permits higher levels of abstraction and improved predictions from data [19]. In medical imaging, the accurate diagnosis of a disease depends on both image acquisition and image interpretation. Thanks to the emerging of modern devices acquiring images very fast and with high resolution, image acquisition has improved substantially over recent years. The image interpretation process, however, has just recently begun to benefit from machine learning.

In our proposed method, by using the characteristics of convolutional neural networks, the MA candidates are selected from the informative part of the image in which the structure is similar to an MA and then a CNN will detect the MA and the non-MA spots. Therefore, our method addresses the imbalanced dataset which is common problem in medical image analysis by using a two-stage training strategy. According to our results, the proposed method can decrease the false-positive rate and can be considered as a powerful solution for automatic MA-detection.

## Methods

**Fig. 1:**
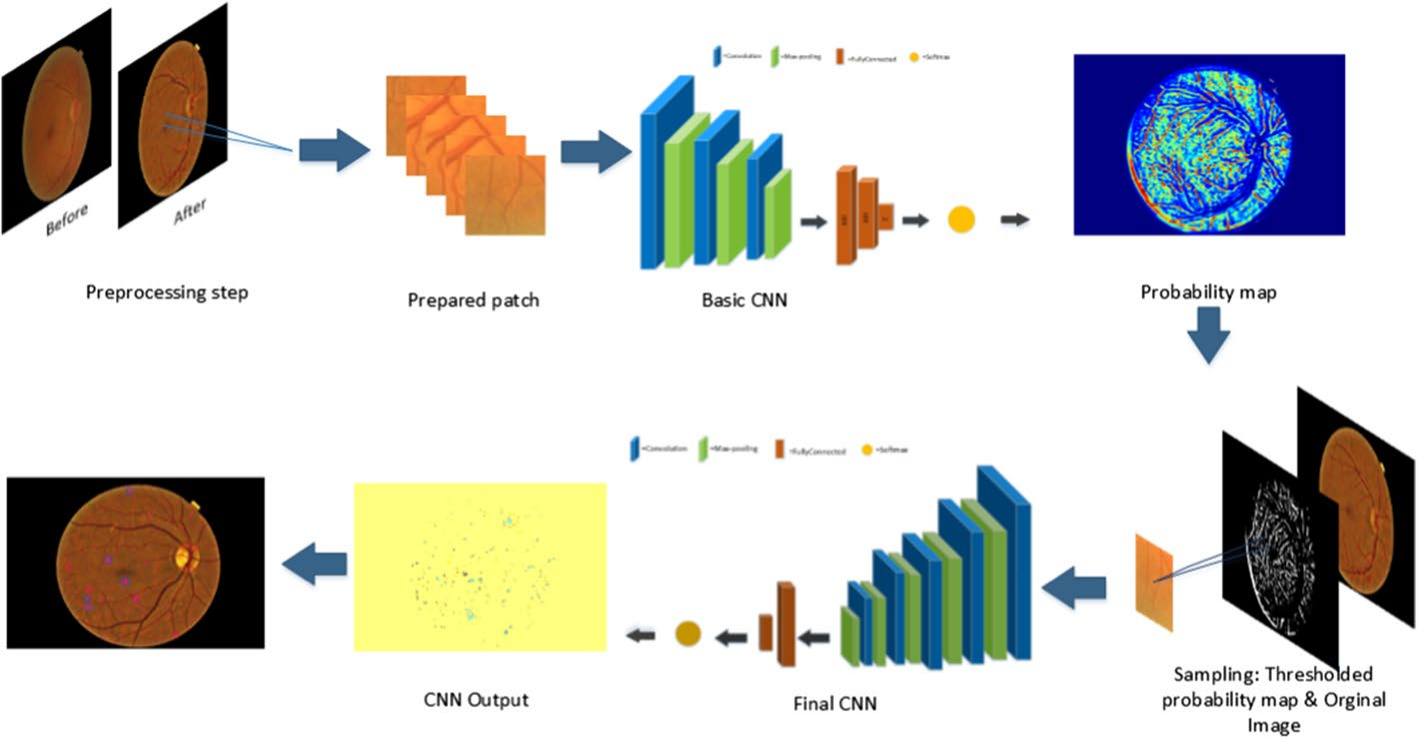
Five steps of the development process of the proposed method. The illustrated fundus images is from E-Ophtha-MA dataset

A schematic representation of our method is depicted in Fig. 1. To address the usual problems of previous works, mentioned in introduction (poor quality of images, the fixed scale of Gaussian kernel, MAs located close to blood vessels and imbalanced dataset), we proposed a two-stage training strategy. First, the pre-processing step is applied then normal samples are selected from a probability map which is the output of the first CNN, called basic CNN. The final CNN classify each pixel in the test images as MA or non-MA. This CNN gets the probability map from the previous stage as the selected samples for the input test images, and result in a final smoothed probability map for each test image showing the probability of being a pixel MA or non-MA. Finally the architectures of CNNs is described.

We have proposed to use two-stage classifiers for MA-detection because of two reasons. First, it is desired to very remove basic false positives using a low cost neural network e.g., basic CNN. And then, use a more complicated/expensive network to classify the remaining pixels. Therefore, it can be computationally very useful. The other reason is that when the classification task would be split into two stages, the second network becomes more expert in handling more difficult examples. Please note that the alternative approach is training of a single network that should handle very hard false-positive cases as well as an enormous number of simple common false-positive cases. This approach is also possible but it is more challenging, it may require online hard example mining, and it is harder to converge. Above all, a significant imbalance in the number of positive and negative samples adds to the complications.

### Pre-processing step

Because the retinal images are usually non-uniformly illuminated, a pre-processing step is needed to apply color normalization and eliminate retina background. This procedure was accomplished by estimating the background image and subtracting that from the original image. The background image was obtained by median filtering the original image with a 30$\times$30 pixel kernel.

Afterwards, input patches with the size of $101\times 101$ were produced from all part of image for training of the basic CNN. This patch size is chosen after examining different sizes ranging [25, 50, 64, 256]. These patches are labeled based on the label of their central pixel from ground truth dataset. Those with a MA pixel at the center are considered as MA samples and those with non-MA pixel are considered as non-MA samples for training.

### Candidate selection by basic CNN

The MA patch is assigned to all windows whose labels are determined by the label of their central pixel; all remaining windows are considered as non-MA class. The result of the “preparing patch” stage contains roughly 29,000 MA instances and 2,58,000 non-MA instances (i.e., approximately 9 times). This issue is called imbalanced data problem which needs special attention. Note that, the largest areas of retinal images are non-vessel and MA structures which are simple to detect; Only a tiny fraction of non-MA samples are hard to classify. Therefore, to detect this tiny fraction of samples we designed a basic CNN. At the first stage of training the basic CNN, an equal number of MA and non-MA patches are selected to train the network to remedy the imbalanced data problem. Because the basic CNN has been trained on a limited fraction of non-MA instances, it tends to classify challenging non-MA instances as MA and will cause a high false-positive rate. Therefore, this output can help to choose challenging patches. The basic CNN output is a probability map specifying the probability of each input pixel belonged to MA. Consequently, we can take advantages of this result to build the balanced input dataset for the final CNN by choosing pixels with the probability greater than 0.6. In fact we built a detector in order to choose informative samples among all non-MAs.

**Fig. 2:**
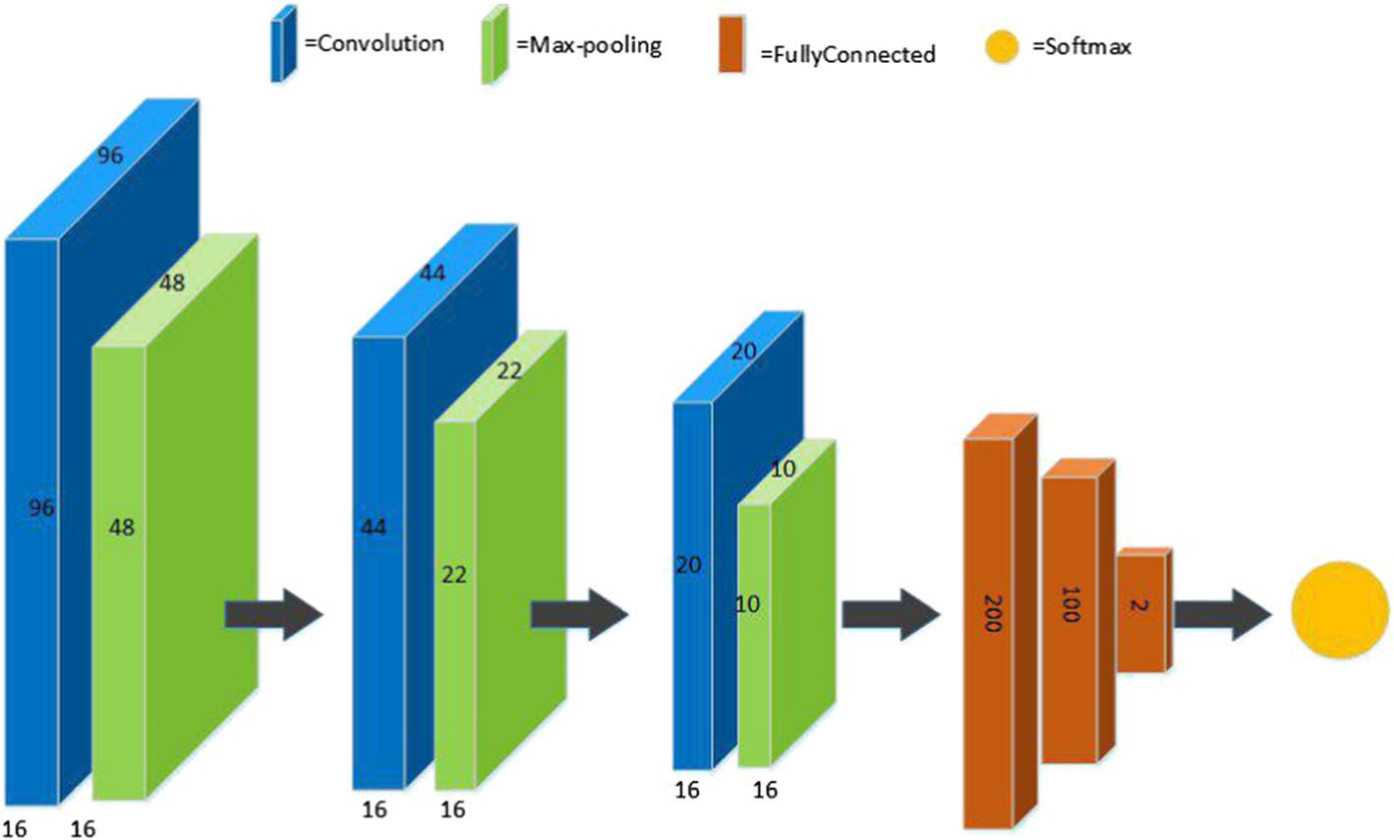
The architecture of basic CNN applied in this project

Figure 2 shows the architecture of basic CNN. The training procedure in CNN is a sequential process that requires multiple iterations to optimize the parameters and extract distinguishing characteristics from images. In each iteration, a subset of samples are chosen randomly and applied to optimize the parameters. This is obtained by back propagation (BP) and minimizing cost function [6].

### Classification by final CNN

The final CNN works as the main classifier to extract the MA candidate regions. This CNN has more layers, and therefore more abstract levels than the basic CNN which lead to a discriminative MA modeling. Unlike the basic CNN which used a random sample from the input dataset pool, the final CNN apply the probability map from the previous stage as the selected samples for the input images. The input samples were obtained by thresholding (T=0.6 which obtained through trial and error) the probability map generated by the first network. This threshold was selected to yield a very high sensitivity and therefore results in many false positives. New patches centered on the pixels of the thresholded probability map were provided as input to the second network for training (Fig. 1).

By using a probability map, we reduced the number of non-MA patches used in training from one million to 258 thousands. Therefore, as the number of patches is reduced to a quarter, the network training time also decreases equally. If the whole images were used in the training stage, most of the training effort would have been wasted and if a uniform sampling were used, although it could have reduced the number of patches, the selected patches would not have been informative samples. So, in our proposed method, we wisely decrease the number of resources required for running the network. In order to do that, we built a concise training dataset by using the probability map to select which patches to feed to the final CNN. For each test image, the output of this CNN is a map which shows the MA-probability of each pixel. However, this map is noisy and a post-processing step is needed.

### Post-processing

In practice, the probability map obtained from the final CNN was extremely noisy. For example when there was two close candidates, they were merged and considered as one. Therefore, to obtain a smoothed probability map, it is convolved with a 5-pixel-radius disk kernel. The local maximum of the new map were expected to lie at the disk centers in the noisy map, i.e., at the centroids of each MA to obtain a set of candidates for each image.

### The architectures of CNNs

Convolutional neural networks (CNNs) is one of the successful type of models for pattern recognition and classification in image analysis. CNN consists of a set of layers called convolutional layers that contains one or more planes as a feature map. Each unit in a plane receives input from a small neighborhood in the planes of the previous layer. Each plane has a fixed feature detector that is convolved with a local window which is scanned over the planes in the previous layer to detect increasingly more relevant image features, for example lines or circles that may represent straight edges or circles, and then higher order features like local and global shape and texture. To detect multiple features, multiple planes are usually used in each layer. The output of the CNN is typically one or more probabilities or class labels [5].

Figure 2 shows one of the architecture of CNN structured we used in MA-detection. As can be seen, the network is designed as a series of stages. The first three stages are composed of convolutional layers (blue) and pooling layers (green) and the output layer (brown) is consist of three fully-connected layers and the last layer is the softmax function.

In this work, two different structures are used for the basic and final CNNs. As can be seen from Fig. 2, the basic CNN includes three convolution layers, each of them followed by a pooling layer, then three fully-connected layers and finally a Softmax layer in the output layer. The final CNN has more layers than the basic CNN. The corresponding layer number of final CNN is five convolution and pooling layers, then two fully-connected and one Softmax classification layer which is fully connected with two neurons for MA and non-MA, see Tables 1 and 2.

**Table 1:** Architectures of final CNN with different input patch-sizes based on trial and error

Layer	Operation	Input size	Detail	Berr, (p)
Layer 1	Input	$3\times 101\times 101$	–	–
Layer 2	Convolutional	$16\times 101\times 101$	$7\times 7$	–
Layer 3	Max pooling	$16\times 50\times 50$	$2\times 2$	0.25
Layer 4	Convolutional	$16\times 48\times 48$	$5\times 5$	–
Layer 5	Max pooling	$16\times 24\times 24$	$2\times 2$	–
Layer 6	Convolutional	$16\times 22\times 22$	$3\times 3$	–
Layer 7	Max pooling	$16\times 11\times 11$	$2\times 2$	0.25
Layer 8	Convolutional	$16\times 10\times 10$	$2\times 2$	–
Layer 9	Max pooling	$16\times 5\times 5$	$2\times 2$	–
Layer 10	Convolutional	$16\times 4\times 4$	$2\times 2$	–
Layer 11	Max pooling	$16\times 2\times 2$	$2\times 2$	–
Layer 12	Fully connected	100	$1\times 1$	–
Layer 13	Fully connected	2	$1\times 1$	–

Berr,(p) is the probability of Bernoulli distribution

**Table 2:** Architectures of basic CNN

Layer	Operation	Input size	Detail	Berr, (p)
Layer 1	Input	$3\times 101\times 101$	–	–
Layer 2	Convolutional	$16\times 96\times 96$	$7\times 7$	–
Layer 3	Max pooling	$16\times 48\times 48$	$2\times 2$	0.25
Layer 4	Convolutional	$16\times 44\times 44$	$5\times 5$	–
Layer 5	Max pooling	$16\times 22\times 22$	$2\times 2$	0.25
Layer 6	Convolutional	$16\times 20\times 20$	$3\times 3$	–
Layer 7	Max pooling	$16\times 10\times 10$	$2\times 2$	0.25
Layer 8	Fully connected	200	$1\times 1$	–
Layer 9	Fully connected	100	$1\times 1$	–
Layer 10	Fully connected	2	$1\times 1$	–

In this work, to increase the accuracy, a dropout training with a maxout activation function is used. Dropout means to reduce over-fitting by randomly omitting the output of each hidden neuron with a probability of 0.25.

Training process is similar to standard neural network using stochastic gradient descent. We have incorporated dropout training algorithm for three convolutional layers and one fully-connected hidden layer. 16 filter sizes $7\times 7$ in the first convolution layer, 16 filter size $5\times 5$ in the second layer, and 16 filter size $3\times 3$ is applied in the third convolution layer, and then maxout activation function is used for all layers in the network except for the softmax layer. The filter size in Max pool layer is $2\times 2$ with stride 2. After each pair convolution and pooling layers, an activation LeakyReLU layer is applied that improved the version of ReLU (rectify linear unit) [34]. In this version, unlike the ReLU in which negative values become zero and so neurons become deactivated, these values in the Leaky ReLU will not be zero, instead, the value of *a* is added to the Eq. 1.

1 $$\begin{aligned} f(x)= {\left\{ \begin{array}{ll} x &{} \quad \text { x}\ge 0\\ a x &{} \quad \text { otherwise} \end{array}\right. } \end{aligned}$$

where *a* is a small constant value (0.01) and *x* is the output of the previous layer. The final layers of the network consist of a fully-connected layer and a final Softmax classification layer. This function produces a score ranging between 0 and 1, indicating the probability of pixel belongs to the MA class. To train the network, loss function of a binary cross entropy is used, note that for a two class system output $t_2=1 - t_1$. Cross entropy calculate the difference between predicted values (p) and targets (t), using the following equation:

2 $$\begin{aligned} L = -t\log (p) - (1-t)\log (1-p) \end{aligned}$$

## Results

To verify our proposed method, we implement the CNNs using deep-learning Keras libraries based on Linux Mint operating system with 32G RAM, Intel (R) Core (TM) i7-6700K CPU and NVIDIA GeForce GTX 1070 graphics card. In this experiment, we used two standard publicly available datasets, Retinopathy Online Challenge [35] and E-Ophtha-MA [36] databases to train and test the proposed method for the detection of MA in retinal images. Retinopathy Online Challenge includes 100 color image of the retina that obtained from Topcon NW 100, Topcon NW 200 and Canon CR5-45NM cameras with JPEG format. The image dimensions are $768\times 576$ , $1058\times 1061$ and $1389\times 1383$ [37]. These images were divided into two parts of 50 subsets of training and testing. However, only the labels of the training set are available. Because the competition website is inactive, which makes it impossible to evaluate our method using the testing set. Consequently, we used cross-validation in the training set to evaluate the method (similar to [28, 38] and [39]). To validate results, the cross-validation is utilized for each dataset separately. By dividing datasets into partitions, then exchange the training and testing sets in successive rounds such that all data have a chance of being trained and tested. E-Ophtha-MA database contains 148 color images with microaneurysm and 233 image with no lesion of JPEG format and with the size of $2544 \times 1696$ and $1440 \times 960$. To have a dataset with equal-size images, the smaller images were resized to the biggest dimension and many patches are extracted from each image. For our training and testing inputs we used about 28786 MA + 258354 Non-MA patches. Moreover, data augmentation is used by mirroring and rotating patches.

For accuracy evaluation, we computed true positive (TP) as the number of MA pixels correctly detected, false positive (FP) as the number of non-MA pixels which are detected wrongly as MA pixels, in other words detected pixels which had no reference of MA within a 5-pixel-radius of our disk kernel, false negative (FN) as the number of MA pixels that were not detected and true negative (TN) as the number of no MA pixels which were correctly identified as non-MA pixels. For better representation of accuracy, sensitivity is defined as follow.

3 $$\begin{aligned} sensitivity=\frac{TP}{TP+FN} \end{aligned}$$

In this experiment, to verify the accuracy of the proposed method, we compared our sensitivity value with the current works (Dashtbozorg [38], chudzik [29], Budak [28], Javidi [40], B Wu [39], Latim [25], OkMedical [10], Waikato group [41], Fujita Lab [18], B Wu’s method [39], Valladolid [42]) on Retinopathy Online Challenge dataset in Table 3 and E-Ophtha-MA dataset in Table 4.

**Fig. 3:**
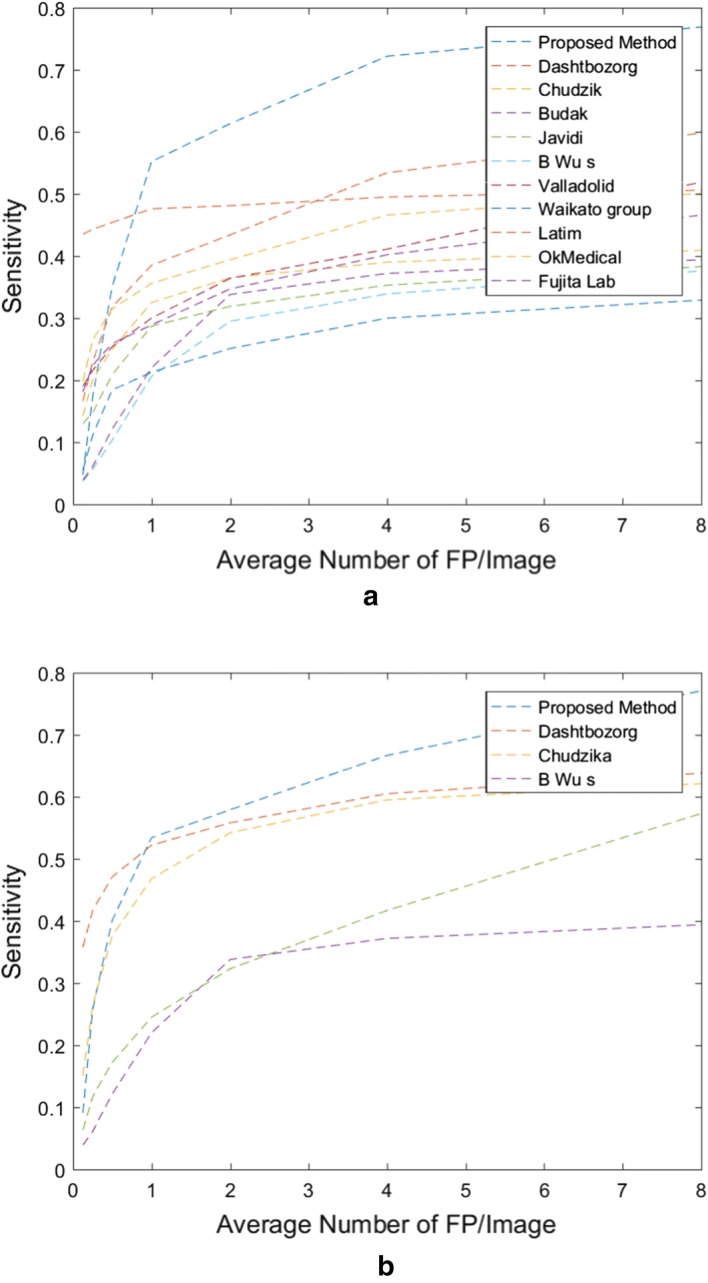
The comparison of free-response receiver operating characteristic curves of the proposed and previous method for **a** Retinopathy Online Challenge dataset and **b** E-Ophtha-MA dataset

In addition, to assess our result, Retinopathy Online Challenge evaluation algorithm [37] is applied and the output of this algorithm is then used to generate a free-response receiver operating characteristic curves that plots the sensitivity against the average number of false-positive detection per image (Fig. 3). These plots, which are extensively used in the literature to estimate the overall performance on this task, represent the per lesion sensitivity against the average number of false-positive detections per image (FPI) obtained on the dataset for different thresholds applied to the candidate probabilities. Thus, free-response receiver operating characteristic curves provide a graphical representation of how the model is able to deal with the detection of true lesions in all the images of the dataset.

Moreover, Table 5 computed the Competition Performance Measure (CPM) as proposed in the Retinopathy Online Challenge [37] and the partial area under the free-response receiver operating characteristic curves ($F_{AUC}$) between 1/8 and 8 FPI to evaluate our results.

## Discussion

**Table 3:** Sensitivities of the different methods in Retinopathy Online Challenge dataset at the various FP/image rates

Free-response receiver operating characteristic results on Retinopathy Online Challenge dataset at average number of False positives per image								
FPs/img								
Sensitivity								
Method	1/8	1/4	1/2	1	2	4	8	Classification method
Proposed method	0.047	0.173	0.351	*0.552*	*0.613*	*0.722*	*0.769*	CNN
Dashtbozorg [38]	*0.435*	*0.443*	*0.454*	0.476	0.481	0.495	0.506	RUSBoost
Chudzik [29]	0.142	0.201	0.250	0.325	0.365	0.390	0.409	CNN
Budak [28]	0.039	0.061	0.121	0.220	0.338	0.372	0.394	DCNN
Javidi [40]	0.130	0.147	0.209	0.287	0.319	0.353	0.383	Discriminative dictionary learning
Wu’s [39]	0.037	0.056	0.103	0.206	0.295	0.339	0.376	KNN
Valladolid [42]*	0.190	0.216	0.254	0.300	0.364	0.411	0.519	GMM
Waikato group [41]*	0.055	0.111	0.184	0.213	0.251	0.300	0.329	Bayesian
Latim [25]*	0.166	0.230	0.318	0.385	0.434	0.534	0.598	Thresholding
OkMedical [10]*	0.198	0.265	0.315	0.356	0.394	0.466	0.501	Dynamic thresholding
Fujita Lab [43]*	0.181	0.224	0.259	0.289	0.347	0.402	0.466	ANN

The quantity given in italic form in each FPs/Img column represents the best result

* Indicate papers which use the full original dataset and others which use the cross-validation technique

**Table 4:** Sensitivities of the different methods in E-Ophtha-MA dataset at the various FP/image rates

Free-response receiver operating characteristic results on E-Ophtha-MA dataset at average number of False positives per image								
Method								
Sensitivity								
FPs/Img	1/8	1/4	1/2	1	2	4	8	Classification method
Proposed method	0.091	0.258	0.401	*0.534*	*0.579*	*0.667*	*0.771*	CNN
Dashtbozorg [38]	*0.358*	*0.417*	*0.471*	0.522	0.558	0.605	0.638	RUSBoost
Chudzika [29]	0.151	0.264	0.376	0.468	0.542	0.595	0.621	CNN
Wu’s [39]	0.063	0.117	0.172	0.245	0.323	0.417	0.573	KNN

The quantity given in italic form in each FPs/Img column represents the best result

From Tables 3 and 4, our proposed method, compared with other methods, has the lowest sensitivity (0.047) when the average number of FP per image (FPs/Img) is 1 / 8, while this value increased quickly and increased to a maximum of 0.769 at FPs/Img equals 8. Dashtbozorg extracted several preliminary MAs candidates by using a gradient weighting technique and an iterative thresholding approach at the first stage. In the next, intensity, shape descriptors and a new set of features based on local convergence index filters is extracted for each candidate. Finally, for the discrimination of the MAs and non-MAs candidates, the collective set of features is trained a hybrid sampling/boosting classifier. While the sensitivity of this method appeared to be high at FPs/Img $<1$, our results are by far higher at FPs/Img $>1$. Chudzik proposed a fully convolutional neural network for detection of microaneurysms including pre-processing and pixel-wise classification and also a fine-tuning procedure called Interleaved Freezing that reduces the amount of time needed to re-train a network. Our sensitivity is higher than this method except at FPs/Img $=1/4,1/8$. Budak used reinforcement sample learning method to train deep convolutional neural network (DCNN). Javidi provided two separate dictionaries, for vessel and non-vessel, which are learned to reconstruct and discriminate information of the retinal image. The proposed method of B Wu’s includes pre-processing, candidate extraction, feature extraction, and KNN classifier. Totally the results of these methods are by far lower than proposed method.

The following methods used original test dataset while above mentioned methods used cross-validation technique due to unavailability of the original dataset. Valladolid assumes all pixels in the image are part of one of three classes: class 1 (background elements), class 2 (foreground elements, such as vessels, optic disk, and lesions), and class 3 (outliers). A three class Gaussian mixture model is fit to the image intensities and a group of MA candidates are segmented by thresholding the fitted model. The sensitivity of this method is 0.190 at FPs/Img $=1/8$ and gradually increase to 0.519 at FPs/Img $=8$. The Waikato group Microaneurysm Detector performs a top-hat transform by morphological reconstruction using an elongated structuring element at different orientations which detects the vasculature. After removal of the vasculature and a microaneurysm matched filtering step the candidate positions are found using thresholding. In comparison with other methods, Waikato group has the lowest sensitivity ranging from 0.055 to 0.329. Latim assumes that microaneurysms at a particular scale can be modeled with 2-D, rotation-symmetric generalized Gaussian functions. It then uses template matching in the wavelet domain to find the MA candidates. Latim method can be considered to have the second high sensitivity value after our proposed method. The sensitivity of this method is 0.166 at FPs/Img $=1/8$ and 0.598 at FPs/Img $=8$. OkMedical responses from a Gaussian filter-bank are used to construct probabilistic models of an object and its surroundings. By matching the filter-bank outputs in a new image with the constructed (trained) models a correlation measure is obtained. In Fujita lab work, a double-ring filter was designed to detect areas in the image in which the average pixel value is lower than the average pixel value in the area surrounding it. Instead, the modified filter detects areas where the average pixel value in the surrounding area is lower by a certain fraction of the number of pixels under the filter in order to reduce false-positive detections on small capillaries. The sensitivity of OkMedical and Fujita ranged from 0.181 to 0.501. Notably, the proposed value which used in a clinical purpose is 1.08 and it provides an indication of “clinically acceptable” FPs/Img, therefore, the system can achieve higher performance for use in a clinical environment [37]. According to this statement our method surpasses other methods at 1.08 point on both Retinopathy Online Challenge and E-Optha-MA datasets by 0.584 and 0.553 respectively.

Figure 3 confirm our results on Tables 3 and 4. This figure shows the free-response receiver operating characteristic , and compare the sensitivity of the proposed method and other methods from [10, 25, 28, 29, 38–43] on Retinopathy Online Challenge and E-Ophtha-MA databases.

From Fig. 3a we can see that the sensitivity of the proposed method on Retinopathy Online Challenge dataset is about 0.2 higher that other methods. It is about 0.6 for the FP greater than 1 and reached the maximum of 0.8, while this number for other methods doesn’t exceed 0.6. The result from Table 3 shows that the sensitivity of proposed method on E-Ophtha-MA dataset increased after FPs/Img $>1$. In addition, Table 5 compares the CPM value and $F_{AUC}$ of the proposed method with the state of the art for both Retinopathy Online Challenge and E-Ophtha-MA datasets. CPM values are 0.461 and 0.471 for Retinopathy Online Challenge and E-Ophtha-MA dataset respectively which is raked in the second place after Dashtbozorg’s scores among state of the art methods. Our results on the images of Retinopathy Online Challenge and E-ophtha-MA dataset achieves $F_{AUC}$ of 0.660 and 0.637 which are significantly higher than the values reported by Dashtbozorg [44].

**Table 5:** Final score (CPM)

Dataset	Method	CPM	$F_{AUC}$
Retinopathy Online Challenge	Proposed method	0.461	*0.660*
	Dashtbozorg [44]	*0.471*	0.484
	Chudzik [9]	0.298	–
	Budak [9]	0.221	–
	Javidi [9]	0.261	–
	B Wu’s [39]	0.202	0.302
	Valladolid [42]	0.322	–
	Waikato group [41]	0.206	0.273
	Latim [25]	0.381	0.489
	OkMedical [10]	0.357	0.430
	Fujita Lab [18]	0.310	0.378
E-Optha-MA	Proposed method	0.471	*0.637*
	Dashtbozorg [44]	*0.510*	0.575
	Chudzik [9]	0.431	–
	Budak [9]	0.431	–
	B Wu’s [39]	0.431	0.386

The quantities given in italic form for “Retinopathy Online Challenge dataset” and “E-Ophtha-MA dataset” represent the best results

Competetion measure (CPM) of Retinopathy Online Challenge at different points

## Conclusion

**Fig. 4:**
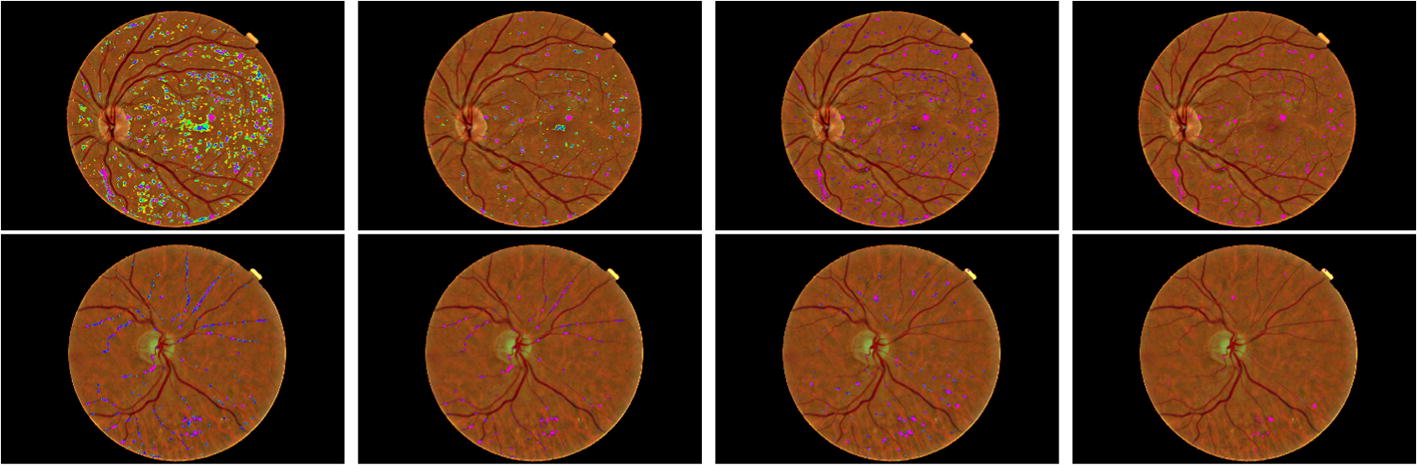
A sample Fundus images of E-Ophtha-MA dataset. Pixel probability maps obtained from the final CNN for a different number of epochs. In initial epochs, the probability map includes low probabilities of MA (depicted as green spots), in the subsequent epochs, the medium and high probabilities are in blue and purple respectively

In this paper, an approach for automatic MA detection in retinal images based on deep-learning CNN is developed to address the previous works problems such as imbalanced dataset and inaccurate MA-detection. In this method, because of using a two-stage CNN, the MAs candidate for classification process are selected from a balanced dataset and informative part of the image where their structure is similar to MA, and this results in decreasing training time. According to our experimental results based on two standard publicly available dataset, the proposed method is about 0.3 higher than other methods. It has a promising sensitivity value of about 0.8 at the average number of false positive per image greater than 6 and can decrease false-positive rate compared to previous methods; it ,therefore, can be considered as a powerful improvement for previous MA-detection based on retinal images approach (Fig. 4)

.

In the proposed method, employing network architecture and network parameters have been developed manually by trial and error, which is a time-consuming and error-prone process. Because of this, nowadays, some autoML methods such as hyper-parameters optimization and neural architecture search (NAS) [45] have been proposed to tackle this problem. These methods can dramatically speed up, improve the design of machine learning pipelines, and tune hyperparameters in a data-driven way. We plan to use the autoML method in our future works. Moreover, we plan to apply this method on other medical application where imbalance data are an issue.

## Data Availability

In this study, two standard publicly available databases, Retinopathy Online Challenge [37] and E-Ophtha-MA [36] databases are used.
